# Does Anterior Disc Displacement with Reduction Affect Postoperative Pain Perception After Root Canal Therapy? A Prospective Comparative Clinical Study

**DOI:** 10.3390/diagnostics16131998

**Published:** 2026-06-26

**Authors:** Burcu Revi, Edanur Maraş, Muhammed Enes Naralan

**Affiliations:** 1 Department of Endodontics, Faculty of Dentistry, Recep Tayyip Erdoğan University, 53100 Rize, Turkey; burcu.revi@erdogan.edu.tr; 2 Department of Oral and Maxillofacial Radiology, Faculty of Dentistry, Recep Tayyip Erdoğan University, 53100 Rize, Turkey; muhammedenes.naralan@erdogan.edu.tr

**Keywords:** disc displacement, endodontics, postoperative pain, root canal therapy, temporomandibular joint, temporomandibular joint disorders

## Abstract

**Background/Objectives**: Temporomandibular disorders may influence the perception of odontogenic pain through shared trigeminal pathways and referred pain mechanisms. This study compared postoperative pain following root canal treatment (RCT) between patients with anterior disc displacement with reduction (ADDwR) and individuals without temporomandibular disorders. **Methods**: The study was registered at ClinicalTrials.gov (NCT07329413; 8 January 2026). Individuals with irreversible pulpitis and symptomatic apical periodontitis in a vital mandibular molar, with or without ADDwR according to the Diagnostic Criteria for Temporomandibular Disorders (DC/TMD), were included. After exclusions and losses to follow-up, 70 patients (35 per group) were analyzed. All RCTs were performed by one clinician using a standardized protocol. Postoperative pain was assessed using the Numeric Rating Scale at 6 and 12 h and on postoperative days 1, 2, 3, 5, and 7. Joint pain and maximum mouth opening were recorded preoperatively and on postoperative day 7, and their associations with postoperative pain were analyzed. **Results**: Patients with ADDwR reported significantly higher pain scores at 6 and 12 h compared with the comparison group (*p* < 0.05). Multivariable analyses showed that ADDwR, female gender, and age were independently associated with postoperative pain at specific time points (*p* < 0.05). Additionally, procedure duration was significantly longer in the ADDwR group than in the comparison group (*p* < 0.05). **Conclusions**: Patients with ADDwR reported higher levels of early postoperative pain following RCT than individuals without temporomandibular disorders. These findings suggest that temporomandibular conditions may influence postoperative pain perception and should be considered when evaluating postoperative pain complaints.

## 1. Introduction

Pain following root canal treatment is defined as endodontic postoperative pain [1]. Previous studies have reported that postoperative pain develops in 2.5% to 60% of patients. Beyond a physiological response, postoperative pain is a clinically important outcome influencing patient satisfaction, confidence in treatment, and quality of life [2]. It represents a multifactorial process affected by biological, technical, and individual factors, including tooth vitality, treatment procedures, clinician experience, and patient-related differences [3]. However, clinicians should also consider that systemic or regional factors may influence postoperative pain perception and reporting. Failure to accurately identify nonodontogenic pain sources may result in misdiagnosis and unnecessary or inappropriate endodontic treatment [4]. Notably, nonodontogenic mechanisms have been reported in a considerable proportion of patients presenting with toothache-like complaints [5].

Temporomandibular disorders (TMDs) are among the most common musculoskeletal conditions associated with nonodontogenic orofacial pain [6]. Disc displacement with reduction (DDwR), an intra-articular temporomandibular joint (TMJ) disorder, accounts for approximately 41% of TMD diagnoses and is also observed in about 33% of asymptomatic individuals [7]. The most prevalent subtype is anterior disc displacement with reduction (ADDwR), in which the articular disc is positioned anterior to the condyle in the closed-mouth position and resumes its normal relationship during mouth opening [8,9]. This condition is commonly associated with clicking sounds during mandibular movements, resulting from transient disc–condyle repositioning [8]. The Diagnostic Criteria for Temporomandibular Disorders (DC/TMD) are a standardized, evidence-based, and widely used diagnostic system with high validity for the classification of TMDs. It provides a structured approach for evaluating pain symptoms related to jaw function, movement, or parafunctional activities and is considered an essential clinical tool in TMD assessment [10].

Previous studies indicate that a considerable proportion of persistent pain following endodontic treatment originates from nonodontogenic sources, occurring in approximately 5–24% of patients [11,12]. Although TMDs are not considered a direct source of odontogenic pain, they have been reported to contribute to pain complaints in approximately 20% of patients seeking endodontic evaluation [13]. Moreover, persistent pain after root canal treatment has been associated with TMDs in approximately 42% of patients [14]. This association may be related to shared neurophysiological mechanisms within the trigeminal system, central sensitization processes, inflammatory responses associated with apical periodontitis, and genetic predispositions [15,16,17,18].

Despite the recognized overlap between TMD-related pain and odontogenic pain, the potential influence of specific TMD subtypes on postoperative pain following root canal treatment remains poorly understood. This knowledge gap is clinically relevant because patients with TMDs frequently present to endodontic clinics with pain complaints, and postoperative pain is often used as an indicator of treatment outcome. A better understanding of whether conditions such as ADDwR are associated with differences in postoperative pain reporting may help clinicians interpret postoperative symptoms more accurately and avoid inappropriate treatment decisions [14,15,16,17,18].

The high prevalence of TMDs and the frequent coexistence of odontogenic and nonodontogenic pain can make differential diagnosis challenging [19]. When pain persists after endodontic treatment in the absence of active dental pathology, underlying muscle- or TMJ-related pain may be overlooked, potentially resulting in misdiagnosis, unnecessary retreatment, or even extraction of otherwise adequately treated teeth [20]. However, the literature review revealed no prospective studies directly comparing postoperative pain experiences following RCT between patients with and without TMDs. Therefore, this study aimed to compare self-reported postoperative pain levels following RCT between patients with ADDwR and individuals without TMD. The null hypothesis was that there would be no statistically significant difference in postoperative pain levels between the two groups.

## 2. Materials and Methods

### 2.1. Study Design and Population

**Figure 1:**
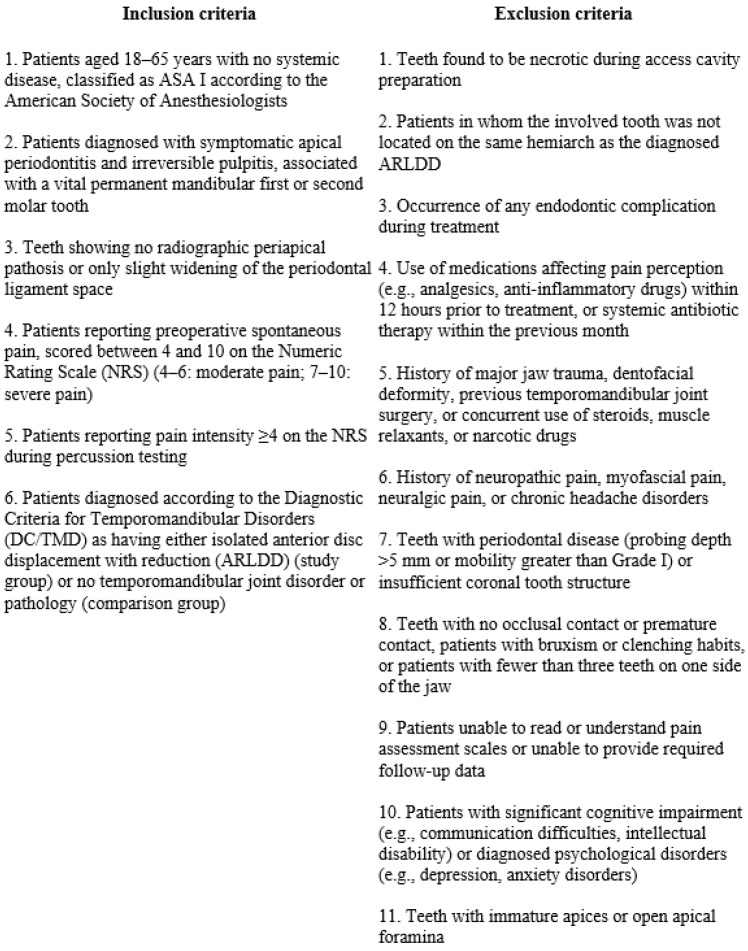
Inclusion and exclusion criteria of patients recruited.

This prospective, quasi-experimental, comparative clinical study was conducted collaboratively at the Departments of Endodontics and Oral and Dentomaxillofacial Radiology. The study was approved by the Institutional Ethics Committee with decision number 2025/82. In addition, the study was registered at ClinicalTrials.gov (National Library of Medicine, National Institutes of Health, USA) under the registration number NCT07329413 on 8 January 2026. Written informed consent was obtained from all patients. Sample size calculation was performed using G*Power software (version 3.1.9.2; Heinrich Heine University, Düsseldorf, Germany), indicating a minimum of 32 participants per group with a 95% confidence level (α = 0.05), a moderate effect size, and 80% statistical power. Participant recruitment was planned to account for potential exclusions and losses to follow-up. Accordingly, 90 participants were enrolled at baseline. The inclusion and exclusion criteria are presented in Figure 1.

### 2.2. Blinding and Study Outline

The endodontic treatment requirements of the patients were evaluated and a TMJ examination was performed by an oral diagnosis and radiology specialist (MEN) with more than 10 years of clinical experience, taking the eligibility criteria into consideration. Participants were classified into two groups according to their temporomandibular status based on the DC/TMD criteria [21]: an ADDwR group and a non-TMD comparison group. At this stage, no information regarding the patient’s TMJ status was disclosed to either the patient or the clinician performing the root canal treatment (BR) (double-blind). Group allocation information was kept concealed until the completion of data analyses (triple-blind).

### 2.3. Assessment

During the clinical evaluation, participants’ sociodemographic characteristics, presence of joint sounds, maximum mouth opening (MMO), percussion sensitivity of the affected tooth, joint pain during jaw function, and preoperative tooth pain scores were recorded.

- Joint sounds were examined using the auscultation method and classified as “present” or “absent.”
- MMO was measured as the distance between the incisal edges of the mandibular and maxillary central incisors using a digital calliper.
- The level of joint pain during jaw function was measured using the Numeric Rating Scale (NRS).
- Percussion sensitivity was measured using the NRS by gently tapping the tooth in a direction parallel to its long axis with the handle of a dental mirror.
- Pain intensity during the preoperative and postoperative follow-up periods was measured using the NRS.

The NRS is an 11-point pain scale ranging from 0 to 10, in which patients rate their pain intensity from 0 (no pain) to 10 (most severe pain) [22]. The NRS is considered a sensitive and standardized measurement tool due to its ease of application in both verbal and written forms and its simple and comprehensible scoring system [23,24]. Therefore, the 11-point NRS, which is widely used for the assessment of postoperative pain and has demonstrated reliability in previous studies, was used in the present study [25,26].

In this study, a structured patient history and clinical examination protocol based on the DC/TMD diagnostic criteria [21] was used to distinguish between disc displacement with and without reduction. The DC/TMD is a standardized, evidence-based, and widely accepted diagnostic system that has demonstrated high validity and reliability for the clinical diagnosis of TMDs [13]. First, participants were asked about the presence of clicking, popping, or rubbing-like sounds in the TMJ region within the past 30 days. The diagnosis of ADDwR was established based on both the patient’s self-report of these sounds and their clinical observation during jaw opening and closing movements. During the clinical evaluation, the presence of characteristic sounds during joint movements was confirmed using a stethoscope and palpation. For the diagnosis of disc displacement without reduction, passively assisted MMO measurements were used as the primary criterion. Each TMJ was evaluated separately, and the obtained clinical findings were classified according to the DC/TMD diagnostic criteria to confirm the diagnosis.

Only vital mandibular first and second molars diagnosed with irreversible pulpitis and symptomatic apical periodontitis were eligible for inclusion. The diagnosis of pulpal and periapical conditions was established in accordance with the diagnostic terminology proposed by the American Association of Endodontists (AAE) [27]. Irreversible pulpitis was diagnosed based on spontaneous pain and/or a prolonged pain response to cold stimulation in vital teeth. Symptomatic apical periodontitis was diagnosed based on sensitivity to percussion in the absence of radiographic periapical pathology. Only teeth fulfilling both diagnostic criteria were included in the study. This approach was adopted to minimize the inclusion of teeth presenting with percussion sensitivity due to non-endodontic conditions, such as occlusal trauma, that may not require root canal treatment. Electric pulp testing (Coxo Medical, Foshan, China) and cold testing (Dispodent, Nadu, India) were used to assess pulp vitality. The contralateral corresponding tooth was used as a reference tooth. Teeth determined to be necrotic upon access cavity preparation were excluded from the study.

### 2.4. RCT Protocol

All endodontic treatments were performed in a standardized manner in a single visit by a single operator (BR) with 6 years of clinical experience. Only one tooth per patient was included in the study.

Inferior alveolar nerve block anesthesia was administered using a 2 mL articaine HCl anesthetic solution containing 1:100,000 epinephrine (Ultracain DS Forte; Pharma Vision, Istanbul, Türkiye). Adequate pulpal anesthesia was clinically verified prior to access cavity preparation based on the absence of pain response to probing and initial dentin cutting, and supplemental anesthesia was administered when required. Following rubber dam isolation, an access cavity was prepared. Root canal working lengths were determined using an electronic apex locator (Woodpex III, Woodpecker, Guilin, China) to be 0.5 mm short of the apical foramen and were confirmed by periapical radiography. Root canals were enlarged up to size 25/0.04 in the mesial canals and 30/0.04 in the distal canals using Endoart Smart Gold files (Inci Dental, Istanbul, Türkiye). If the use of these files resulted in insufficient apical instrumentation, the root canals were enlarged up to a maximum of two sizes larger according to the canal length and anatomy. After completion of root canal preparation, final irrigation was performed using 5 mL of 17.5% ethylenediaminetetraacetic acid (EDTA), 5 mL of saline solution, and 5 mL of 2.5% sodium hypochlorite (NaOCl) solution. Subsequently, the root canals were obturated using the lateral condensation technique with a resin-based root canal sealer (ADSeal; Meta Biomed, Cheongju, South Korea). Following the application of adhesive with Gluma Bond Universal (Heraeus Kulzer GmbH, Hanau, Germany), the canal orifices were sealed with a flowable composite (Nova Compo HF, Imicryl, Konya, Türkiye). A permanent restoration was performed using composite resin (Nova Compo C, Imicryl, Konya, Türkiye). The treatment was completed after occlusal adjustment.

### 2.5. Outcome Measures

For the evaluation of postoperative pain, each patient was given a pain follow-up form (NRS) to record their pain levels at 6, 12, 24, 48, and 72 h, as well as on postoperative days 5 and 7 after RCT. Patients were instructed on how to record their pain levels using the NRS and were reminded to document their pain scores at predefined time points. Ibuprofen 400 mg was prescribed for use in cases of severe pain. To minimize the potential masking effect of analgesic consumption on pain assessment, patients who reported postoperative analgesic use were excluded from the final analysis. A follow-up appointment was scheduled one week later. On postoperative day 7, MMO was re-evaluated using a digital calliper, joint sounds were assessed by auscultation, and joint pain and percussion sensitivity of the treated tooth were evaluated using the NRS.

### 2.6. Statistical Analysis

The primary analyses were performed using R version 4.5.0 (R Foundation for Statistical Computing, Vienna, Austria). Additional multivariable regression analyses were conducted using IBM SPSS Statistics version 23 (IBM Corp., Armonk, NY, USA) and NCSS version 11 (NCSS, LLC, Kaysville, UT, USA). Normal distribution was examined using the Shapiro–Wilk test. For comparisons of quantitative data with normal distribution between two independent categorical variables, the independent two-sample *t* test was used; for comparisons of quantitative data without normal distribution, the Mann–Whitney U test was used. Yates’ correction, Monte Carlo-corrected Fisher’s exact test, and Fisher’s exact test were used to examine associations between categorical variables. For comparisons of quantitative data with normal distribution between two dependent categorical variables, the paired-sample *t* test was used; for comparisons of quantitative data without normal distribution, the Wilcoxon signed-rank test was used. The Friedman test was used for time-based comparisons of three or morerepeated measurements. Multiple comparisons were examined using the Dunn test. The McNemar test was used for comparisons of dependent categorical variables. The effects of independent variables on the dependent variable were assessed using linear regression models when the dependent variable met the assumption of normality, whereas robust regression models were applied when the dependent variable was not normally distributed. Descriptive statistics for quantitative variables were presented as mean ± standard deviation and median (minimum–maximum). For categorical variables, frequency, percentage, and *n* (%) were reported. The level of significance was set at *p* < 0.05.

## 3. Results

**Figure 2:**
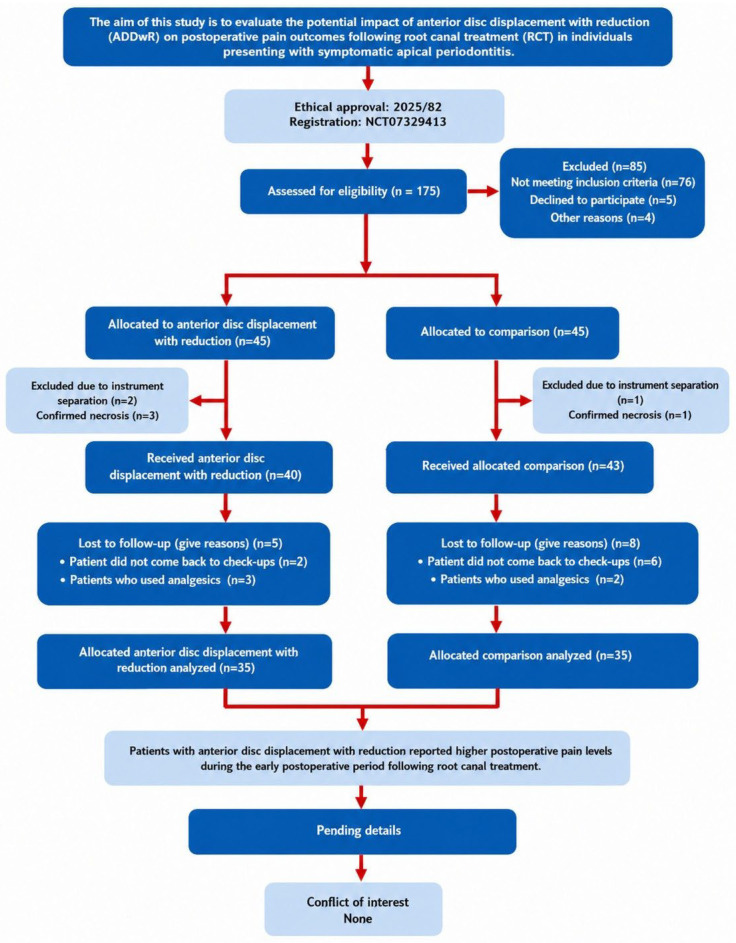
A flow diagram of the study.

After exclusions and attrition, 70 participants (35 per group) completed the study and were included in the final analysis (Figure 2).

### 3.1. Demographic Data and Procedure Duration

The mean procedure duration was 71 min in the ADDwR group and 63.86 min in the comparison group, and the procedure duration was found to be significantly longer in the ADDwR group (*p* < 0.05). No significant differences were detected between the groups in terms of age, gender, marital status, education level, and tooth number (*p* > 0.05; Table 1).

### 3.2. Clinical Evaluation

In the intergroup comparison, the preoperative joint pain level in the ADDwR group was found to be statistically significantly higher compared to the comparison group (*p* < 0.05). However, no significant difference was detected between the two groups on postoperative day 7 (*p* > 0.05). In both intra-group and intergroup comparisons, no statistically significant difference was observed between the ADDwR and comparison groups in terms of preoperative and postoperative MMO values (*p* > 0.05). In addition, while no significant difference was found between the ADDwR and comparison groups in terms of preoperative and postoperative percussion sensitivity (*p* > 0.05), postoperative sensitivity levels were found to be significantly reduced compared to preoperative values in both groups (*p* < 0.001; Table 2). The presence of preoperative and postoperative joint sounds was found to be statistically significantly higher in the ADDwR group compared to the comparison group (*p* < 0.05; Table S1).

### 3.3. Comparison of Postoperative Pain Scores

Pain scores in the ADDwR group were significantly higher than those in the comparison group at 6 and 12 h and on postoperative day 1 (*p* < 0.05; Figure S1). In within-group comparisons, postoperative pain scores in both groups tended to decrease over time starting from 6 and 12 h (*p* > 0.05; Table 3; Figure S2). When postoperative pain levels were compared between the 18–30 and 31–65 age groups, pain scores were significantly higher in the 31–65 age group only on postoperative day 7 (*p* < 0.05), while no significant differences were observed at other time points.

### 3.4. Multivariable Analysis of Factors Associated with Postoperative Pain

Multivariable regression analyses were performed to evaluate factors independently associated with postoperative pain. After adjustment for demographic and clinical variables, ADDwR remained significantly associated with higher postoperative pain scores at 6 and 12 h postoperatively (*p* < 0.05; Table 4 and Table 5, respectively). Female gender was also significantly associated with higher pain scores at 6 h, 12 h, and postoperative day 1 (*p* < 0.05). On postoperative days 2 and 3, age was independently associated with postoperative pain (*p* < 0.001). No significant association was observed between preoperative joint pain and postoperative pain (*p* > 0.05; Tables S2–S4). Regression analyses for postoperative days 5 and 7 were not feasible because of the large proportion of zero pain scores.

## 4. Discussion

During the RCT process, patient comfort and the accurate identification and management of postoperative pain are critical for patient satisfaction and long-term treatment success [28]. TMDs are among the major nonodontogenic causes of orofacial pain, and their variable clinical presentations and frequent overlap with other types of orofacial pain complicate the diagnostic process [6,29]. One of these disorders, ADDwR, although often asymptomatic, may cause pain in some cases and be confused with odontogenic pain [10,30,31]. Therefore, several studies emphasize that nonodontogenic factors such as TMDs should be considered in the evaluation of endodontic postoperative pain [32,33]. In this context, investigating the potential relationship between ADDwR and postoperative pain following RCT may contribute to differential diagnosis and help prevent unnecessary dental interventions [32]. Accordingly, the aim of this study was to investigate the association between ADDwR and self-reported postoperative pain following RCT by comparing patients with ADDwR and individuals without TMD.

The results showed that patients with ADDwR reported significantly higher postoperative pain scores at 6 and 12 h and on postoperative day 1. Accordingly, the null hypothesis for these time points was rejected. Furthermore, multivariable regression analyses demonstrated that ADDwR remained independently associated with higher postoperative pain scores at 6 and 12 h after adjustment for baseline joint pain and other demographic and clinical variables. This finding suggests that the observed differences in postoperative pain cannot be explained solely by the higher preoperative joint pain levels observed in the ADDwR group. This observation supports a potential association between ADDwR and altered postoperative pain perception or reporting during the early healing period following RCT [13,30,32].

In within-group comparisons, postoperative pain levels decreased over time in both the ADDwR and comparison groups. However, no significant differences were observed between the groups on postoperative days 2, 3, 5, and 7. This finding suggests that differences in postoperative pain reporting between patients with and without ADDwR were most pronounced during the early postoperative period and became less evident over time.

To date, no clinical studies have directly assessed the association between ADDwR and endodontic postoperative pain perception, which limits direct comparison with the present findings. Nevertheless, several studies have examined the relationship between TMDs and odontogenic pain and indirectly support the findings of the present study [31,32]. Daline et al. [13] reported the prevalence of painful TMDs of 54% in patients presenting for endodontic treatment and noted that TMDs contributed to the primary pain complaint in 20% of cases. Similarly, Baptista [31] reported a prevalence of painful TMDs of 89.5% in patients requiring endodontic treatment and reported an association between tooth pain and TMDs in 57.9% of patients. Nixdorf et al. [12] reported that pain was TMD-related in 42% of patients with persistent pain after RCT. In addition, Signorelli et al. [32] found a significant relationship between the number of endodontically treated teeth and the side of the jaw affected by TMD symptoms.

Previous studies suggest that both peripheral and central sensitization can occur in individuals with TMDs [34,35]. A meta-analysis reported significantly increased mechanical pain sensitivity in patients with TMDs, including the trigeminal region, with more pronounced differences observed in individuals with moderate to severe symptoms compared to asymptomatic cases [35]. Although most DDwR cases remain asymptomatic, some individuals develop joint inflammation and pain [36]. Moreover, studies have reported increased joint sensitivity even in asymptomatic DDwR cases [37]. Consistent with these findings, the present study showed significantly higher preoperative joint pain levels in individuals with ADDwR compared to the comparison group, while no difference was observed on postoperative day 7. However, the higher preoperative joint pain observed in the ADDwR group may reflect a temporary increase in joint tenderness associated with ADDwR rather than persistent pain in the TMJ over time [32].

One possible explanation is that patients with ADDwR may exhibit alterations in pain processing or increased sensitivity within the trigeminal system, which could contribute to differences in postoperative pain perception following endodontic procedures. However, the mechanisms underlying the observed association remain unclear [15,16,17,18]. Although ADDwR remained independently associated with higher early postoperative pain scores after adjustment for measured confounders, residual confounding cannot be excluded. Therefore, future prospective studies incorporating comprehensive assessments of pain sensitivity, psychosocial factors, central sensitization, and broader TMD characteristics are needed to better clarify the pathways underlying the association between ADDwR and postoperative pain following root canal treatment.

Few studies have investigated the effect of procedure duration on postoperative pain, and the direction of this relationship remains unclear [38,39]. Mikesell et al. [40] reported greater postoperative pain following longer procedures (≥45 min), whereas Claffey et al. [41] reported increased intraoperative pain with longer procedures, which was attributed to reduced local anesthetic effectiveness and increased anxiety. In the present study, procedure duration was significantly longer in individuals with ADDwR than in the comparison group. While the reasons underlying this difference remain unclear, clinical characteristics associated with TMDs, including difficulties related to prolonged mouth opening, may have contributed [42]. However, procedure duration was not associated with postoperative pain levels. Therefore, although longer treatment duration may theoretically contribute to patient discomfort, the higher postoperative pain scores observed in the ADDwR group cannot be explained solely by differences in procedure duration. Similarly, MMO was not associated with postoperative pain in either between-group or within-group comparisons, which may be explained by comparable preoperative MMO values among participants.

Although gender distribution did not differ between groups, multivariable analyses demonstrated that female gender remained independently associated with higher postoperative pain scores at 6 and 12 h and on postoperative day 1. These findings are consistent with previous studies reporting a higher incidence and severity of postoperative pain among females [43,44]. Sex-related differences in postoperative pain experience may be related to hormonal influences, cognitive–emotional factors, and neurophysiological variations [45].

Similarly, age was independently associated with postoperative pain on postoperative days 2 and 3. Although the mechanisms underlying this association remain unclear, age-related differences in tissue healing, inflammatory responses, and neural processing may contribute to variations in postoperative pain experience [46]. Because the present study was not designed to investigate these mechanisms, further studies are needed to clarify the factors underlying the observed association between age and postoperative pain.

Among intra-articular TMJ disorders, DDwR accounts for approximately 41% of clinical TMD diagnoses and has been reported in about 33% of asymptomatic individuals [7]. Although disc displacement may occur in anterior, posterior, lateral, or medial directions, posterior and purely lateral displacements are rare, with ADDwR representing the most common form [9]. Accordingly, the present study focused on ADDwR, the most prevalent form of disc displacement in individuals with TMD. Postoperative pain after RCT has been reported to occur more frequently in the mandibular arch, particularly in molar teeth. Several explanations have been proposed, including the dense trabecular structure of mandibular bone, which may compromise regional blood circulation and delay healing, as well as the complex multirooted anatomy of mandibular molars that may hinder adequate canal disinfection [43,46]. In addition, pain associated with ADDwR may be perceived as referred ear or tooth pain and may mimic symptoms originating from mandibular or maxillary molars because of shared trigeminal innervation [33,47]. Accordingly, to ensure sample standardization, only mandibular first and second molars were included in the present study.

In patients presenting for endodontic treatment, teeth most strongly associated with TMDs have been reported to be those with symptomatic apical periodontitis exhibiting percussion sensitivity and referred pain [13]. Increased sensitivity in the trigeminal somatosensory system and disturbances in pain modulation may contribute to the simultaneous occurrence of referred pain conditions observed in conjunction with TMJ symptoms [13]. In painful teeth, increased pulpal levels of neuropeptides such as calcitonin gene-related peptide (CGRP), substance P (SP), and neurokinin A contribute to peripheral sensitization [48]. An observational study has suggested that central sensitization may contribute to the exacerbation of pre-existing or subclinical TMD-related pain following pulpal injury [32]. Furthermore, pulpitis and apical periodontitis sustain inflammatory processes associated with TRPV1 activation and increased neuropeptide release, which may enhance neural sensitization and contribute to the persistence of orofacial pain [17,49]. Therefore, only teeth diagnosed with irreversible pulpitis and symptomatic apical periodontitis, with vital pulp and no radiographic evidence of periapical changes, were included in the present study.

The prospective design of the present study and the inclusion of a comparison group without TMD represent important methodological strengths. Given that endodontic postoperative pain perception may be influenced by multiple biological and clinical factors, rigorous standardization of the study protocol is essential [50]. Accordingly, only vital permanent teeth fulfilling the diagnostic criteria for both irreversible pulpitis and symptomatic apical periodontitis were included, and the included tooth types were standardized across participants. All endodontic treatments were performed by a single experienced clinician using standardized materials and a predetermined treatment protocol, while clinical assessments and postoperative pain follow-up were conducted using standardized methods. Potential confounding factors were addressed through both strict eligibility criteria and multivariable analyses adjusting for demographic and clinical variables. In addition, individuals using medications that could influence inflammatory responses or pain perception, as well as those with systemic diseases, were excluded from the study. This approach was adopted to minimize the potential influence of pharmacological agents on postoperative pain reporting during the follow-up period and to ensure a more accurate assessment of postoperative pain [51,52]. However, because analgesic use may be more common among individuals experiencing greater pain, this approach may have resulted in some underestimation of postoperative pain levels.

However, the present study has certain limitations. Since pain is an individual and subjective experience, reported pain scores may vary among individuals depending on factors such as pain threshold, psychological status, previous pain experiences, and ways of expressing pain. This variability may limit the homogeneity of the results due to differing responses to the same clinical stimulus [53]. In addition, despite the prospective design of the study, a causal relationship between ADDwR and postoperative pain cannot be established because of its observational and non-randomized nature. Although several potential confounders were included in the regression models, residual confounding cannot be excluded. Therefore, the findings should be interpreted with caution. In addition, the diagnosis of ADDwR was based on the DC/TMD protocol, which relies on clinical examination and patient history; magnetic resonance imaging (MRI), the reference standard for assessing disc position, was not used. Although the DC/TMD has demonstrated high validity and reliability, some degree of diagnostic misclassification cannot be excluded, and asymptomatic or subclinical TMD cases may have remained undetected. Therefore, the results should be interpreted with this limitation in mind [7,32]. Furthermore, future studies including a broader range of tooth types and regions potentially affected by referred TMJ pain may help improve the generalizability of these observations.

## 5. Conclusions

Patients with ADDwR reported higher early postoperative pain scores following root canal treatment than individuals without TMD. After adjustment for demographic and clinical variables, ADDwR remained independently associated with higher postoperative pain scores at 6 and 12 h. Female gender and age were also independently associated with postoperative pain at specific postoperative time points. These findings suggest that temporomandibular conditions should be considered when evaluating postoperative pain symptoms following endodontic treatment. Future studies incorporating broader TMD subtypes, comprehensive assessments of pain sensitivity, psychosocial factors, and MRI-confirmed diagnoses are needed to further clarify the relationship between ADDwR and postoperative pain.

## Figures and Tables

**Table 1:** Comparison of demographic characteristics and procedure time between ADDwR and comprasion groups.

	ADDwR	Comparison	Total	Test Statistic	*p*
Procedure time	71 ± 14.49	63.86 ± 10.01	67.43 ± 12.87	2.400	**0.020** ^x^
Age	27 (18–59)	30 (18–58)	30 (18–59)	493.000	0.162 ^y^
Gender					
Female	23 (65.7)	21 (60)	44 (62.9)	0.061	0.805 ^z^
Male	12 (34.3)	14 (40)	26 (37.1)		
Marital status					
Single	16 (45.7)	14 (40)	30 (42.9)	0.058	0.809 ^z^
Married	19 (54.3)	21 (60)	40 (57.1)		
Education Level					
Primary school	6 (17.1)	10 (28.6)	16 (22.9)	3.015	0.392 ^t^
High school	14 (40)	8 (22.9)	22 (31.4)		
Associate degree	4 (11.4)	6 (17.1)	10 (14.3)		
University degree	11 (31.4)	11 (31.4)	22 (31.4)		
Tooth Number					
First molar	21 (60)	24 (68.6)	45 (64.3)	0.249	0.618 ^z^
Second molar	14 (40)	11 (31.4)	25 (35.7)		

^x^ Independent samples *t*-test; ^y^ Mann–Whitney U Test; ^z^ Yates correction, ^t^ Fisher Exact test with Monte Carlo correction; ADDwR: Anterior disc displacement with reduction. Bold was used to highlight statistically significant value.

**Table 2:** Comparison of preoperative and postoperative clinical parameters between ADDwR and comprasion groups.

	ADDwR	Comparison	Total	Test Statistic	*p*
Preoperative joint pain	1.51 ± 2.42	0.2 ± 1.02	0.86 ± 1.96	790.500	**0.003 ^x^**
	0 (0–8)/40.59	0 (0–6)/30.41	0 (0–8)		
Postoperative joint pain	0.86 ± 1.54	0.49 ± 1.4	0.67 ± 1.47	698.500	0.161 ^x^
	0 (0–6)	0 (0–6)	0 (0–6)		
Test statistic	86.500	8.000			
*p*	0.138 ^z^	0.351 ^z^			
Preoperative MMO	43.21 ± 7.21	44 ± 6.73	43.6 ± 6.93	−0.471	0.639 ^y^
	44 (29–57.5)	43 (31.3–60.6)	43.6 (29–60.6)		
Postoperative MMO	43.52 ± 8.39	44.87 ± 6.72	44.2 ± 7.58	574.500	0.660 ^x^
	42.7 (28.7–60.1)	42 (36.6–64.4)	42.05 (28.7–64.4)		
Test statistic	−0.461	210.000			
*p*	0.648 ^t^	0.087 ^z^			
Preoperative percussion	6.6 ± 1.75	7.06 ± 1.49	6.83 ± 1.63	513.000	0.237 ^x^
	6 (4–10)	7 (4–10)	7 (4–10)		
Postoperative percussion	1.69 ± 2.19	0.91 ± 1.52	1.3 ± 1.91	741.500	0.096 ^x^
	1 (0–10)	0 (0–6)	0 (0–10)		
Test statistic	617.500	630.000			
*p*	**<0.001 ^z^**	**<0.001 ^z^**			

^x^ Mann–Whitney U test; ^y^ Independent samples *t*-test; ^z^ Wilcoxon signed-rank test; ^t^ Paired samples *t*-test. Values are presented as mean ± standard deviation and median (minimum–maximum). ADDwR: Anterior disc displacement with reduction; MMO: Maximum mouth opening. Bold was used to highlight statistically significant values.

**Table 3:** Comparison of postoperative pain scores over time within and between the ADDwR and comparison groups.

	ADDwR	Comparison	Total	Test Statistic	*p*
Postoperative Pain 6 h	4.4 ± 3.21 ^a^	2.8 ± 2.72 ^a^	3.6 ± 3.06	793.500	**0.032** ^x^
	4 (0–10)	2 (0–9)	3 (0–10)		
Postoperative Pain 12 h	3.83 ± 2.94 ^a^	2.34 ± 2.68 ^ab^	3.09 ± 2.89	797.000	**0.028** ^x^
	4 (0–10)	2 (0–9)	2.5 (0–10)		
Postoperative Pain Day 1	2.69 ± 2.71 ^b^	1.43 ± 1.88 ^bc^	2.06 ± 2.4	784.000	**0.037** ^x^
	2 (0–9)	0 (0–8)	2 (0–9)		
Postoperative Pain Day 2	2.06 ± 2.5 ^bc^	1.03 ± 1.65 ^cd^	1.54 ± 2.16	753.500	0.080 ^x^
	1 (0–9)	0 (0–8)	1 (0–9)		
Postoperative Pain Day 3	1.09 ± 1.8 ^cd^	0.54 ± 1.2 ^de^	0.81 ± 1.54	730.500	0.110 ^x^
	0 (0–8)	0 (0–6)	0 (0–8)		
Postoperative Pain Day 5	0.83 ± 1.69 ^d^	0.37 ± 1.09 ^e^	0.6 ± 1.43	695.500	0.206 ^x^
	0 (0–7)	0 (0–6)	0 (0–7)		
Postoperative Pain Day 7	0.57 ± 1.69 ^d^	0.09 ± 0.28 ^e^	0.33 ± 1.22	672.500	0.229 ^x^
	0 (0–9)	0 (0–1)	0 (0–9)		
Test statistic	109.714	112.263			
*p*	**<0.001 ^y^**	**<0.001 ^y^**			

^x^ Mann–Whitney U test; ^y^ Friedman Test; Data are presented as mean ± standard deviation and median (minimum–maximum). ^a–d^: Within-group comparisons: time points sharing the same letter do not differ significantly within the ADDwR group; ^a–e^: time points sharing the same letter do not differ significantly within the healthy group. ADDwR: Anterior disc displacement with reduction, h: Hours. Bold was used to highlight statistically significant values.

**Table 4:** Multivariable linear regression model for postoperative pain at 6 h.

Variable	*β*^1^ (95% CI)	SE	*β* ^2^	*t*	*p*-Value	Zero-Order	Partial	Part	VIF
Intercept	−1.679 (−9.468 to 6.109)	3.894	—	−0.431	0.668	—	—	—	—
Group (ADDwR)	1.669 (0.134 to 3.205)	0.768	0.275	2.174	**0.034**	0.257	0.270	0.245	1.257
Female sex	2.438 (0.669 to 4.207)	0.884	0.388	2.757	**0.008**	0.558	0.335	0.310	1.558
Tooth number (#36)	1.198 (−0.293 to 2.690)	0.746	0.189	1.607	0.113	0.090	0.203	0.181	1.090
Preoperative joint pain	−0.279 (−0.714 to 0.155)	0.217	−0.179	−1.286	0.204	0.524	−0.164	−0.145	1.524
Preoperative maximum mouth opening	−0.015 (−0.143 to 0.112)	0.064	−0.035	−0.243	0.809	0.638	−0.031	−0.027	1.638
Age	0.013 (−0.059 to 0.086)	0.036	0.043	0.366	0.715	0.096	0.047	0.041	1.096
Preoperative pain	−0.117 (−1.356 to 1.122)	0.619	−0.061	−0.189	0.851	0.073	−0.024	−0.021	8.073
Preoperative percussion sensitivity	0.080 (−1.142 to 1.302)	0.611	0.043	0.131	0.896	0.067	0.017	0.015	8.372
Procedure duration	0.042 (−0.019 to 0.103)	0.030	0.177	1.386	0.171	0.221	0.176	0.156	1.283

Adjusted *R*^2^ = 0.125; Durbin–Watson = 2.031; F = 2.095, *p* = 0.044; *R*^2^ = 0.239; ADDwR, anterior disc displacement with reduction; CI, confidence interval; SE, standard error; VIF, variance inflation factor. *β*^1^: Unstandardized regression coefficient; standardized *β*^2^: Standardized regression coefficient. Bold was used to highlight statistically significant values.

**Table 5:** Multivariable linear regression model for postoperative pain at 12 h.

Variable	*β*^1^ (95% CI)	SE	*β* ^2^	*t*	*p*-Value	Zero-Order	Partial	Part	VIF
Intercept	1.318 (−6.073 to 8.709)	3.695	—	0.357	0.723	—	—	—	—
Group (ADDwR)	1.51 (0.053 to 2.967)	0.728	0.263	2.072	**0.043**	0.257	0.258	0.235	1.257
Female sex	1.777 (0.098 to 3.456)	0.839	0.299	2.118	**0.038**	0.558	0.264	0.240	1.558
Tooth number (#36)	1.267 (−0.149 to 2.682)	0.708	0.212	1.790	0.078	0.090	0.225	0.203	1.090
Preoperative joint pain	−0.148 (−0.561 to 0.265)	0.206	−0.100	−0.717	0.476	0.524	−0.092	−0.081	1.524
Preoperative maximum mouth opening	−0.058 (−0.179 to 0.062)	0.060	−0.140	−0.967	0.338	0.638	−0.124	−0.11	1.638
Age	0.028 (−0.041 to 0.097)	0.035	0.095	0.801	0.426	0.096	0.103	0.091	1.096
Preoperative pain	0.247 (−0.929 to 1.422)	0.588	0.135	0.420	0.676	0.073	0.054	0.048	8.073
Preoperative percussion sensitivity	−0.306 (−1.466 to 0.854)	0.580	−0.173	−0.528	0.599	0.067	−0.068	−0.060	8.372
Procedure duration	0.021 (−0.037 to 0.078)	0.029	0.092	0.718	0.476	0.221	0.092	0.081	1.283

Adjusted *R*^2^ = 0.114; Durbin–Watson = 1.801; F = 1.989, *p* = 0.056; *R*^2^ = 0.230. ADDwR, anterior disc displacement with reduction; CI, confidence interval; SE, standard error; VIF, variance inflation factor. *β*^1^: Unstandardized regression coefficient; standardized *β*^2^: Standardized regression coefficient. Bold was used to highlight statistically significant values.

## Data Availability

The raw data supporting the findings of this study are available from the corresponding author upon reasonable request due to ethical and privacy considerations related to participant data.

## Supplementary Materials

The following supporting information can be downloaded at: https://www.mdpi.com/article/10.3390/diagnostics16131998/s1, Table S1: Comparison of joint sound presence between ADDwR and comprasion groups; Table S2: Results of the linear regression analysis for postoperative pain on day 1; Table S3: Results of the linear regression analysis for postoperative pain on day 2; Table S4: Results of the linear regression analysis for postoperative pain on day 3; Figure S1: Time-dependent median distribution of postoperative pain scores in the groups; Figure S2: Box plots of pain scores measured over time.
