# Supplementary material for: Does Anterior Disc Displacement with Reduction Affect Postoperative Pain Perception After Root Canal Therapy? A Prospective Comparative Clinical Study
Source: Diagnostics (Basel). 2026 Jun 26;16(13):1998. doi: 10.3390/diagnostics16131998 (PMC13359714; doi:10.3390/diagnostics16131998)
Supplement: Supplementary file 1 [file diagnostics-16-01998-s001.zip › diagnostics-4360417-supplementary.pdf]

Table S1. Comparison of joint sound presence between ADDwR and comprasion groups.

	ADDwR	Comprasion	Total	Test Statistic	*p*
Preoperative joint sound					
Absent	7 (20)	35 (100)	42 (60)	43,393	**<0.001**^{x}
Present	28 (80)	0 (0)	28 (40)		
Postoperative joint sound					
Absent	6 (17.1)	35 (100)	41 (58.6)	46,156	**<0.001**^{x}
Present	29 (82.9)	0 (0)	29 (41.4)		
p	1.000^y	--			

^{x} Yates correction ^{y} McNemar test; n (%); ADDwR: Anterior disc displacement with reduction.

Table S2. Results of the linear regression analysis for postoperative pain on day 1.

Variable	β^1 (95% CI)	SE	β^2	*t*	*p*-value	Zero-order	Partial	Part	VIF
Intercept	-1.682 (-7.751 to 4.387)	3.034	—	-0.554	0.581	—	—	—	—
Group (ADDwR)	1.181 (-0.015 to 2.378)	0.598	0.248	1.975	0.053	0.257	0.247	0.221	1.257
Female sex	1.853 (0.475 to 3.232)	0.689	0.376	2.69	0.009	0.558	0.328	0.301	1.558
Tooth number (#36)	0.485 (-0.678 to 1.647)	0.581	0.097	0.834	0.407	0.090	0.107	0.093	1.090
Preoperative joint pain	-0.076 (-0.415 to 0.263)	0.169	-0.062	-0.448	0.656	0.524	-0.058	-0.05	1.524
Preoperative maximum mouth opening	-0.02 (-0.119 to 0.080)	0.05	-0.057	-0.397	0.692	0.638	-0.051	-0.044	1.638
Age	0.035 (-0.021 to 0.092)	0.028	0.146	1.249	0.217	0.096	0.159	0.14	1.096
Preoperative pain	-0.003 (-0.968 to 0.963)	0.483	-0.002	-0.006	0.995	0.073	-0.001	-0.001	8.073
Preoperative percussion sensitivity	-0.047 (-0.999 to 0.906)	0.476	-0.032	-0.098	0.922	0.067	-0.013	-0.011	8.372
Procedure duration	0.027 (-0.020 to 0.074)	0.024	0.144	1.137	0.260	0.221	0.145	0.127	1.283

F=2.206; *p*=0.034; R^2=0.249; Adj. R^2=0.136; Durbin Watson=1.804; ADDwR, anterior disc displacement with reduction; CI, confidence interval; SE, standard error; VIF, variance inflation factor. β^1: Unstandardized regression coefficient; standardized β^2: Standardized regression coefficient.

Table S3. Results of the linear regression analysis for postoperative pain on day 2.

Variable	β^1 (95% CI)	SE	β^2	*t*	*p*	VIF
Intercept	-1.773 (-5.309 to 1.764)	1.768	—	-1.239	0.220	—
Group (ADDwR)	0.152 (-0.545 to 0.849)	0.349	0.059	0.524	0.602	1.260
Female sex	0.714 (-0.089 to 1.517)	0.402	0.276	2.190	0.032	1.554
Tooth number (#36)	0.288 (-0.390 to 0.965)	0.339	0.107	1.019	0.313	1.082
Preoperative joint pain	0.140 (-0.058 to 0.337)	0.099	0.200	1.631	0.108	1.469
Preoperative maximum mouth opening	0.010 (-0.048 to 0.068)	0.029	0.052	0.405	0.687	1.625
Age	0.051 (0.018 to 0.084)	0.017	0.403	3.811	<0.001	1.096
Preoperative pain	0.289 (-0.273 to 0.852)	0.281	0.365	1.227	0.225	8.674
Preoperative percussion sensitivity	-0.452 (-1.007 to 0.103)	0.277	-0.580	-1.915	0.060	9.002
Procedure duration	0.017 (-0.010 to 0.045)	0.014	0.174	1.549	0.127	1.235

F = 4.229, $p<0.001$; $R^2 = 0.388$; Durbin–Watson = 1.772. ADDwR, anterior disc displacement with reduction; CI, confidence interval; SE, standard error; VIF, variance inflation factor. β^1: Unstandardized regression coefficient; standardized β^2: Standardized regression coefficient.

Table S4. Results of the linear regression analysis for postoperative pain on day 3.

Variable	β^1 (95% CI)	SE	β^2	*t*	*p*-value	VIF
Intercept	-0.474 (-1.538 to 0.591)	0.532	—	-0.890	0.377	—
Group (ADDwR)	0.193 (-0.021 to 0.407)	0.107	0.216	1.808	0.076	1.278
Female sex	-0.021 (-0.259 to 0.217)	0.119	-0.023	-0.177	0.860	1.546
Tooth number (#36)	0.168 (-0.042 to 0.377)	0.105	0.175	1.600	0.115	1.077
Preoperative joint pain	0.051 (-0.009 to 0.111)	0.030	0.222	1.695	0.095	1.540
Preoperative maximum mouth opening	0.000 (-0.018 to 0.018)	0.009	0.001	0.009	0.993	1.667
Age	0.023 (0.013 to 0.033)	0.005	0.496	4.483	<0.001	1.096
Preoperative pain	-0.091 (-0.264 to 0.082)	0.086	-0.327	-1.053	0.297	8.663
Preoperative percussion sensitivity	0.069 (-0.106 to 0.243)	0.087	0.248	0.787	0.435	8.893
Procedure duration	-0.002 (-0.010 to 0.006)	0.004	-0.056	-0.472	0.639	1.251

Model statistics: F = 3.301, $p = 0.003$; $R^2 = 0.331$; Durbin–Watson = 1.798. ADDwR, anterior disc displacement with reduction; CI, confidence interval; SE, standard error; VIF, variance inflation factor. β^1: Unstandardized regression coefficient; standardized β^2: Standardized regression coefficient.

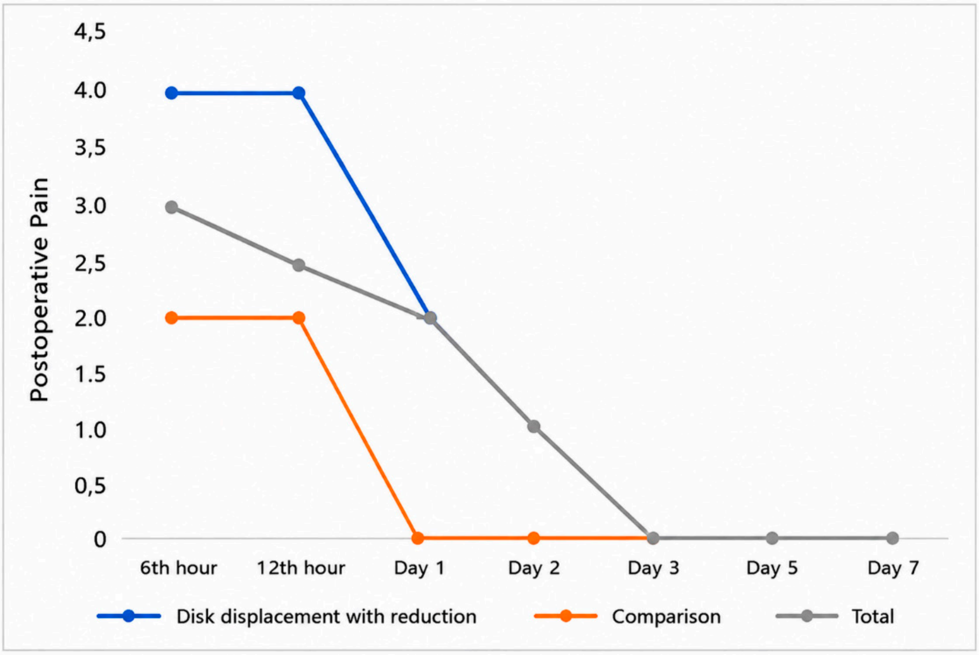

Figure S1. Time-dependent median distribution of postoperative pain scores in the groups.

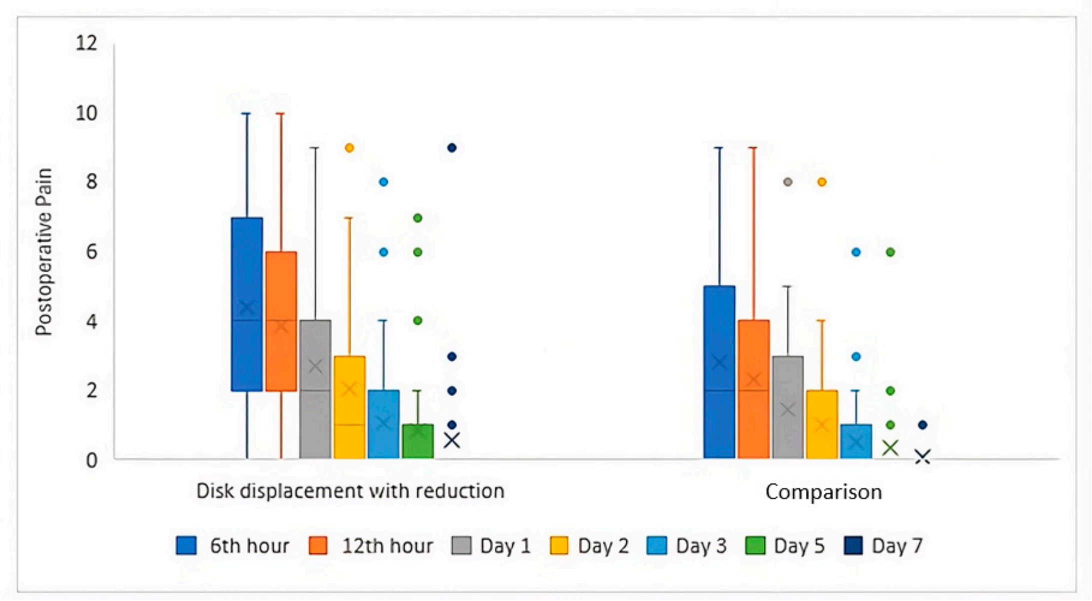

Figure S2. Box plots of pain scores measured over time.
